# A multidimensional framework for rating health system performance and sustainability: A nine plus one ranking system

**DOI:** 10.7189/jogh.11.04025

**Published:** 2021-05-08

**Authors:** Laura Müller, Reida El Oakley, Mohammed Saad, Ali H Mokdad, Giamal A Etolhi, Antoine Flahault

**Affiliations:** 1 Institute of Global, Health, Faculty of Medicine, University of Geneva, Switzerland; 2 Cardiac Centre, King Abdel Aziz Specialist Hospital, Taif, Saudi Arabia; 3 The Libyan International Medical University, Benghazi, Libya; 4 Institute for Health Metrics and Evaluation, University of Washington, Seattle,Washington, USA

## Abstract

**Background:**

Health Care provision in terms of prevention, detection and treatment is primarily dependent on the quality of the hosting Health System. In its health report 2000, the WHO's attempt to assess and rank health systems’ quality Worldwide was heavily criticized. We propose a novel framework for health system performance and ranking using three indicators for three domains; general health system performance, clinical outcome of treatment applied to the main causes of death and health system sustainability domains.

**Methods:**

Each domain was rated as “A – high”, “B – intermediate” or “C – poor” according to the aggregate score values of its three indicators. Hence the highest rank a health system can achieve is “AAA” and the lowest is “CCC”. If there is a need to define a “numerical rank” to further differentiate health systems with similar rating from one another, the total health expenditure per capita per year was used as an additional “number 10” indicator to achieve that level of differentiation. The framework was applied to Health Systems serving most of the World population including China, India, Brazil, USA, Russia, Germany, Japan, UK, France, Singapore and Switzerland. Data pertinent to each indicator was captured from published reports in peer-reviewed journals and/or from official websites. A Delphi survey was conducted for data not available online.

**Results:**

Among the 11 health systems tested, no one scored AAA, Switzerland, France, Germany and Japan scored AAB, Singapore scored ABB, UK scored BBB, USA, Russia and China scored BBC, Brazil scored BCC while India scored CCC. Total health expenditure per capita per year lead to ranking Switzerland first followed by France, Germany, and Japan.

**Conclusion:**

This novel ranking system is a practical and an applicable tool that test health system performance and sustainability. It can be utilized to guide all organizations, people and actions whose primary intent is to promote, restore or maintain health to achieve their targets. An International Health System Ranking database that will be hosted by the Institute of Global, Health, Faculty of Medicine, University of Geneva, Switzerland.

The United Nations (UN) and the World Health Organization (WHO) favored Universal Health Coverage (UHC) – defined as “…achieving the desired outcome of health system performance, whereby all people who need health services (promotion, prevention, treatment, rehabilitation and palliation) receive them, without undue financial hardship…” – as an overarching goal for sustainable development in Health [1,2]. Most countries, regardless of their income levels, are now engaged in health care reforms efforts to extend the spectrum of UHC service packages provided to individuals, in combination with maximum possible financial protection [2,3]. The spectrum and quality of UHC offered, however, will primarily be influenced by the quality of the hosting health system [4,5] that is defined as “all organizations, people and actions whose primary intent is to promote, restore or maintain health” [6]. The same applies to implementation of International Health Regulation to combat epidemics/pandemics.

The WHO have previously ranked health systems [6] using five indicators derived from what was considered to be the main goals of a health system: (1) Health (level and distribution), (2) Responsiveness (level and distribution) and (3) Financial fairness. Although considered the best health system ranking available by a few [7-9], the WHO ranking was heavily criticized and has not been universally accepted [10-13]. The main criticism is due to the use of a limited set of general indicators and none-use of direct clinical (case-mix) outcome indicators that led to condemning health systems renowned for its clinical excellence such as those in North America to a relatively lower rank and elevating health systems with far less clinical service capabilities to higher ranks. The Canadian and the United States’ Health Systems were ranked 30^th^ and 37^th^, respectively despite the fact that the outcome of many clinical services in Canada and the United State are amongst the best in the World. Moreover, low-income countries may also be disadvantaged by the WHO ranking, which does not properly assess the efforts a country could make to improve access to care for a portion of its poorest population, if this improvement implies an increase in inequalities within the country. Indeed, the health distribution indicator measures relative differences in quality, without taking into account the absolute level of quality [14]. We have also noted that total expenditure on health, which is a major factor in health systems performance and sustainability, was not fairly employed in the WHO 2000 report as it was not selected as the 6^th^ indicator. Non-use of “total expenditure on health” as an indicator in the WHO 2000 report has deprived many better-funded health systems from achieving better ranks. The rationales for indicators selection in the WHO 2000 report, quality of data and the techniques used for its interpretation and analysis were also subject to major criticism [10-13], even if its methodology was considered the most complete and transparent compared to others existing rankings (Bloomberg; Commonwealth Fund ranking) [7]. Furthermore, Health System sustainability was not previously addressed in any of the Health Systems Ranking reports [7,9,15].

Despite these highly critical reviews, however, the World Health Report 2000 has certainly placed health systems performance on the political agenda and stimulated major programs in health system research and analysis [7,9].

The wealth of health systems data generated since 2000 through the WHO, the Health Metrics Network, the Institute for Health metrics and Evaluation, the European Observatory on Health Systems and Policies, and others [16-20] have prompted health systems researchers, clinicians and politicians to re-emphasize the need for “…*identifying simple, practical, and understandable ways to assess the health systems performance with its complex interlinked dimensions*…” [4,7,9,15].

In this manuscript, we propose a novel conceptual multidimensional framework for rating health system performance using 9 general, clinical, and sustainability outcome indicators. This score has the advantage of being simple, without complicated statistical calculations. Our framework allows rating Health Systems ranging from AAA (Excellent) and CCC (Poor). If there is a need to “numerically rank” different health systems with a similar rating, the total health expenditure per capita per year may be used as a tool to achieve that level of differentiation. This framework may be used as a tool to accredit various health systems.

## METHODS

We propose the use of nine health system outcome indicators categorized under the following three domains;

1. General performance (**Table 1**),

**Table 1:** General performance indicators (Domain I**)**

	Target performance (score = 3)	Towards target performance (score = 2)	Far below target performance (score = 1)
1. UHC effective coverage (% of the population)	>90%	50%-90%	<50%
2. Female’s HALE at birth (in years)	>70	50-70	<50
3. Number surgical procedures performed as % of the minimum number needed*	>90%	50%-90%	<50%
General performance score	9	6	3

UHC – universal health coverage, HALE – healthy life expectancy

*Using region-specific denominators.
2. Clinical outcome (**Table 2**), and

**Table 2:** Major clinical performance indicators (Domain II)

	Target performance (score = 3)	Towards target performance (score = 2)	Far below target performance (score = 1)
4. Overall HAQ index	>90	50-90	<50
5. HAQ index for Hodgkin’s’ lLymphoma	>90	50-90	<50
6. % 30 d-survival of patients hospitalized with acute MI	>95%	90%-95%	<90%
Clinical performance score	9	6	3

HAQ – health access and quality, MI – myocardial infarction
3. Sustainability (**Table 3**).

**Table 3:** Health system equity and sustainability indicators (Domain III)

	Target performance (score = 3)	Towards target performance (score = 2)	Far below target performance (score = 1)
7. % of accredited hospitals providing tertiary care	>90%	50%-90%	<50%
8. Financial protection, health care equity and funding sustainability (% of the population)	HSAs* for >90% or Out of Pocket expenditure on health care* less than <5% of total household income	HSAs^)^ for 50%-90% or Out of Pocket expenditure on health care* of 5%-7 · 5% of total household income	HSAs* for <50% or Out of Pocket expenditure* on health care of >7.5% of total household income
9. Gov. funding for health research (% of GDP)	>0.2%	0.05%-0.2%	<0.05%
Health system equity and sustainability score	9	6	3

HSAs – health savings accounts, GDP – gross domestic product

*Includes policyholder's share of health insurance costs like contributions levied on the total gross income or health insurance premium.

The first domain, which includes general indicators, was designed to assess the well-being of a population. The second domain measures the competencies and clinical performance of health services in a country. The last domain focuses on the sustainability of a health system in terms of who finances it and how, but also in terms of investments made to promote research in health care. The exact reasons for choosing each indicator are summarized in the discussion.

The outcome indicators were selected and differ from those proposed in the WHO 2000 report according to the following criteria;

- Consistently represent the status of the relevant domain through:
  - Assessment of attaining general health system goals as defined by the WHO,
  - Evaluating the outcome of therapeutic interventions as for the main causes of death, including coronary artery disease or cancer,
  - Testing health system sustainability via health care funding mechanism(s), infrastructure and human resources development as well as funding levels of health research;
- With evidence published in peer-reviewed journals (or obtainable) for most (if not all) countries;
- Least influenced by non-health system policies;
- Simple, non-composite and without complicated statistical calculations, when possible;
- Amenable for a numerical scoring.

We tested our framework for rating health system performance by selecting 11 countries most of which are high-income countries. We believed that high-income countries would have more available and/or accessible data.

Data were reviewed based on evidence published in peer-reviewed journals or from official websites (ie, who.int, oecd.org or official government websites). We always used the latest data available. Results remains simple without any statistical calculation. Only Indicators #3 and #8 was obtained by combining data from two different data sources.

Some of the data was difficult to identify, so we therefore conducted a Delphi survey. The principle of a Delphi survey is to ask a group of individuals to estimate the values of the missing data. We didn’t impose any selection criteria on our survey participants other than being an expert in public health and working for one of the 11 selected countries. We invited 27 experts (2 experts per country on average) to participate in our survey and we asked them to assess the level of performance of the countries for which we had not found all the data. The survey was conducted in two rounds. The first round consisted in submitting an anonymous questionnaire through an online platform; the second round consisted in analyzing the responses and sharing the results with the experts. The most frequently answered score was adopted. If there was a need to differentiate two scores with similar rate of answers, the most favorable score was selected. The more unanimous (100%) the score awarded, the more reliable it was considered to be**.**

### Weights assigned to indicators

Target Health System Performance was considered to exceed 90% of optimum results for an individual indicator from data published in peer-reviewed journals. Each of the nine indicators is assigned a score “weight” of “3” if it achieves that target performance level eg, if the Health Access and Quality (HAQ) index for an individual cause of death within that system is >90, a score “2” if it achieves close to the target performance eg, if the HAQ index for an individual cause of death is 50-90, and a score of “1” if its achievements is far below target performance level eg, if the HAQ index for an individual cause of death is less than 50.

## RESULTS

### Overall System Rating and Ranking

The collective score of the three indicators in every domain is given a rate of A, B or C as follows (**Table 4**):

**Table 4:** Overall health rating and ranking

	Grade A	Grade B	Grade C
General performance indicators (1-2-3)	8-9	5-7	3-4
Major clinical performance indicators (4-5-6)	8-9	5-7	3-4
Health system sustainability indicators (7-8-9)	8-9	5-7	3-4

1. “A” if the total score for its three indicators is 8-9, or
2. “B” if the total score for its three indicators is 5-7, or
3. “C” if the total score for its three indicators is 3-4.

Health Systems was then rated as AAA, BBB, CCC, or any combination of As, and/or Bs, and/or Cs (**Table 5**). The rate of “C” in any domain is an indication of a failing (CCC and BCC) or critical (BBC, ABC and AAC) health system performance, and implies an urgent need for a comprehensive health system review. Stable health system (BBB, ABB) rating indicates that Health System performance is adequate, yet requires attention to indicators scoring two or less, and mandates a review of the relevant domain(s). Above average (AAB) and excellent (AAA) ratings indicates the health system is of high quality and is poised to sustain economic, political, natural or man-made crisis. If there is a need to define a “numerical rank” to further differentiate health systems with similar rating from one another, the total health expenditure per capita per year was used as an additional (#10) indicator to achieve that level of differentiation.

**Table 5:** Overview of rating and raking scores

	Score for individual indicator									Total score for individual domain			Overall rating	Total health expenditure per capita per year in US$ [21]	Numerical rank*
	**1**	**2**	**3**	**4**	**5**	**6**	**7**	**8**	**9**	**I**	**II**	**III**			
Switzerland	3 [22]	3 [23]	3 [19,24]	3 [18]	3 [18]	2 [25]	3 [26]	1 [27]	3 [28]	9	8	7	AAB	9956.3	1
France	3 [22]	3 [23]	3 [19,24]	3 [18]	2 [18]	2 [25]	3 [29]	3	3	9	7	9	AAB	4379.9	2
Germany	2 [22]	3 [23]	3 [19,24]	3 [18]	3 [18]	2 [25]	2	1 [30]	3	8	8	6	AAB	5033.5	3
Japan	3 [22]	3 [23]	3 [19]	3 [18]	3 [18]	2 [25]	2	2	2	9	8	6	AAB	4169.0	4
Singapore	3 [22]	3 [23]	2 [19]	3 [18]	2 [18]	1 [25]	2	3 [31]	1 [32]	8	6	6	ABB	2618.7	5
UK	2 [22]	2 [23]	3 [19,24]	2 [18]	3 [18]	2 [25]	1	2	2 [32]	7	7	5	BBB	3858.7	6
USA	2 [22]	2 [23]	3 [19,24]	2 [18]	3 [18]	2 [25]	2	1	1	7	7	4	BBC	10246.1	7
Russia	2 [22]	2 [23]	3 [19,33]	2 [18]	2 [18]	1	1	2	1 [32]	7	5	4	BBC	585.9	8
China	2 [22]	3 [23]	2 [19,24]	2 [18]	1 [18]	1	1	2	2	7	4	5	BBC	440.8	9
Brazil	2 [22]	2 [23]	3 [19,33]	2 [18]	1 [18]	1	1	1	1	7	4	3	BCC	928.8	10
India	1 [22]	2 [23]	1 [19]	1 [18]	1 [18]	1	1	1	1	4	3	3	CCC	69.3	11

*Prioritized primarily according to the overall rating and secondarily according to the total health expenditure per capita per year.

### Interpretation and integration of the Delphi method results

Among the 11 tested health systems, the Swiss Health System was ranked first followed by France, Germany and Japan (**Table 5**). Several data came from the Delphi survey since not all were available in the literature (**Table 6**). Indicators #1 #2, #4, and #5 could be found entirely in the literature (in peer-reviewed journals), unlike indicators #7 or #8, which had to be estimated mostly by the Delphi survey. Data could be found for the remaining indicators, but in a very heterogeneous manner depending on the country, which is why the Delphi study had to be used on a case-by-case basis. For example, less data was found for India, China or Brazil. While indicator #6 is well documented by OECD (Organization for Economic Co-operation and Development) and its member countries, it is less well reported at the global scale. Data for indicator #9 are also very incomplete depending on the country even if extracted from the WHO Global Observatory on Health Research and Development (R&D). A good understanding of health system implementation (eg, hospital accreditation processes), a detailed description regarding the distribution of health costs (eg, household participation in health care costs through insurance premiums or deductibles) was sometimes necessary to rate countries adequately, which is why we preferred to use the Delphi survey for countries where we were uncertain (indicator #7 and #8). The data found on these indicators (#7 #8) came mostly from official government websites and not from global database.

**Table 6:** Overview of Delphi survey results*

	Score for individual indicator				
	**3**	**6**	**7**	**8**	**9**
Switzerland					
France				3, 71,4%	3, 71,4%
Germany			2, 71.4%		3, 71.4%
Japan	3, 57.1%		2, 85.7%	2, 71.4%	2, 71.4.7%
Singapore	2, 57.1%		2, 57.1%		
UK			1, 57.1%	2, 57.1%	
USA			2, 85.7%	1, 100%	1, 57.1%
Russia		1, 71.4%	1, 71.4%	2, 57.1%	
China		1, 71.4%	1, 71.4%	2, 57.1%	2, 57.1%
Brazil		1, 71.4%	1, 71.4%	1, 85.7%	1, 85.7%
India	1, 85.7%	1, 85.7%	1, 85.7%	1, 100%	1, 85.7%

## DISCUSSION

The analysis associated with the development of this alternative score is based on an academic and neutral perspective. The rationale for the selection of each indicator is presented in a discussion supported by documented data sources.

A key element of this score was to circumvent the limitations of the World Health Report 2000, for the reasons mentioned in the introduction. In order to do so, we have elected to:

1. Increase the number of performance indicators to a total of nine indicators used to rate health systems + one additional indicator (total health expenditure per capita per year) to allow numerical ranking of health systems that may have similar rating
2. Include outcome indicators for clinical services including mortality-to-incidence ratios (MIRs) and risk-standardized outcome of therapeutic interventions targeting major causes of Death
3. Include outcome indicators for long term sustainability of health system performance, including sustainability during economic crises, Natural and/or Man-made disasters, endemics or pandemics as well as political upheavals
4. Elaborate on the criteria and the rational used in selecting individual indicator as well as the methods and reasons for weighing each indicator in a relatively simple, practical and understandable framework
5. Select independent - rather than composite - indicators to avoid potential technical errors in calculating the latter.
6. Weigh these indicators using either data published in peer-reviewed scientific journals or data that can be obtained without much difficulty for most – if not all – countries

The first indicator of our score considers the proportion of a population that has access to the essential health services they need, when and where they need them, without getting into financial difficulty (UHC) [34]. The term “essential” is used to describe the health services that a country considers to be available to all people who need them [3]. However, the contents of these health services vary between different health systems and are often focused on primary and secondary health care services such as access to Vaccinations and the WHO’s essential medicines list. Advanced, often costly, tertiary and quaternary health service packages such as advanced care for the mentally ill, emergency medical and surgical services, interventions for coronary artery disease, immunotherapy for cancer, bone marrow and organ transplantation are rarely included in today’s UHC packages [1-3,35]. Measuring effective coverage at the health-system level is therefore essential for understanding whether health services are of quality and can produce health gains for populations of all ages. Indicator 1# was derived from the UHC effective coverage index based on the Global Burden of Diseases, Injuries, and Risk Factors Study (GBD) 2019. The GBD (2019) assessed UHC effective coverage for 204 countries and territories from 1990 to 2019 by incorporating health needs in term of – promotion, prevention, treatment, rehabilitation, and palliation – among different population age group, what showed its utility and role in supporting improved health outcomes for all people [22].

With increasing life expectancy in most countries, the question of whether the additional years of life gained are spent in good health or poor health has been increasingly relevant. Moreover, the burden of disabling conditions has serious implications for health system planning and health-related expenditures [17]. In this work, we considered the number of years of life spent in good health and especially among women essential to assess the performance of a health system (indicator #2). Female’s Healthy life expectancy (HALE) at birth provides a summary measure for woman health across by considering all causes combined and by weighting years lived with a measure of functional health loss before death. It’s the most comprehensive measure among competing expectancy metrics [17,36].

Surgical care is essential for managing diverse health conditions – such as injuries, obstructed labour, malignancy, infections and cardiovascular disease – and an indispensable component of a functioning health system. All health systems do not provide these essential interventions to their population [19,24]. Recent surveys and expert opinions suggest that up to 30% of the global burden of disease requires a minimum number of surgical procedures for adequate management [19,37]. Data from the Global Burden of Disease (GBD) study from the Institute of Health Metrics and Evaluation estimated that more than 321 million surgical procedures would have been needed to address surgically-amenable diseases in the 6.9 billion World population in 2010 [19]. The minimum number of surgical procedures required ranged between 3383 operations per 100 000 population in central Latin America and 6495 operations per 100 000 in Western sub-Saharan Africa. Only robust health system will cater-for and be ready to deliver such complex and relatively costly, yet cost-effective, services. This indicator is measured as the ratio between the number of surgeries performed on average per country and the estimated surgical procedure needed by region of the world. This estimated surgical procedure need by regions and not by countries can lead to bias, since there may be significant differences between countries within a same region. However, at the current time we do not have more specific data per country that allows this analysis.

A key component of achieving universal health coverage is ensuring that all populations have not only access to health care but also to quality health care. Indicators #3 and #4 assess the concept of predictable and avoidable mortality in the presence of effective care. This is an essential starting point for assessing the quality of care provided by health services. They were derived from the Healthcare Access and Quality (HAQ) Index report that was based on a systematic analysis of 32 diseases from which death is preventable (ie, coronary heart disease, cancer, major trauma) in the presence of effective care, using health system data from 195 countries between 1990 and 2016 [18].

That analysis involved the following steps:

- Constructing MIRs for cancers and risk-standardizing non-cancer deaths to remove variations in mortality not directly amenable to health care,
- Calculating the HAQ Index on the basis of principal components analysis, providing an overall score of personal health-care access and quality on a scale of 0-100; Zero was determined by the first percentile observed (ie, highest death rates or MIRs), and 100 was applied to the 99^th^ percentile (ie, lowest death rates or MIRs).

This indicator was also chosen because it indirectly tests the ability for collaboration between health services within the country and with neighboring countries. For example, in order to manage the high influx of patients with COVID-19, Swiss, French or German hospitals transferred their patients to relieve hospitals on the verge of saturation and to guarantee quality care [38]. Finally, we incorporated the HAQ index for Hodgins lymphoma as an independent indicator. Hodgkin lymphoma is a highly curable disease by current treatment standards. It implies access to diagnostic and treatment modalities that may not be available in settings with restricted health care resources [39]. A good score on this indicator therefore directly measures access to quality care. It also specifically tests the ability of a medical service to function as a multidisciplinary team with multiple interdisciplinary discussions within a hospital, a fundamental criterion for quality care. Taking into account the contributions of different health professionals is fundamental to improve quality of care but also to provide a comprehensive patient-centered care and a good use of resources [40,41].

Cardiovascular disease is the leading cause of death in the world: more people die each year from cardiovascular disease than from any other cause [42]. The capacity to manage a myocardial infarction seemed to us essential to score the performance of a health system. This indicator measures not only the performance of a hospital (presence of a catheterization room or trained specialists, for example) but also the timeliness of access to this care through a competent care network [43,44]. These data are still undocumented as data on only 7 out of 11 countries were found. The data were obtained mainly for OECD countries.

A hospital must be certified by the appropriate authorities and integrated into the health system to which it belongs in order to function in an efficient and sustainable way. By harmonizing hospital standards with those of other institutions and levels of care, continuity of care is ensured and the health care network can be strengthened [45-47]. In addition, with international patient mobility, the need for transparency in health care, pressure from patient associations to guarantee quality care, the legalization of medicine but also medical tourism and the accreditation of health care services seems to us essential to ensure the quality of care in a long-term perspective. In this work, we wish to emphasize the fact that not only that the accreditation of hospitals is essential to deliver long term quality services, but that it should be carried out by a systematic procedure through external and independent organisms (ie, JCI accreditation [48]).

Testing Governments’ and systems’ Commitments to funding continuous professional development of health care workers and health care facilities is also achieved through indicators #7, since hospital accreditation is intended to certify that health care workers meet quality requirements for working in the facility. Funding on health and research by an individual Government is met with indicator 9, with the objective that they commit at least 0.2% of its Gross Domestic Products [20].

The economic crisis brought an unprecedented attention to the issue of health system sustainability in the developed world. The discussion, however, has been mainly limited to “traditional” issues of cost-effectiveness, quality of care, and, lately, patient involvement. Not enough attention has yet been paid to the issue of who pays and, more importantly, to the sustainability of financing. This fundamental concept in the economics of health policy needs to be reconsidered carefully. In a globalized economy, as the share of labor decreases relative to that of capital, wage income is increasingly insufficient to cover the rising cost of care. At the same time, as the cost of Social Health Insurance through employment contributions rises with medical costs, it imperils the competitiveness of the economy [15,31]. In this score we highlight the health funding model through Health Savings Accounts (HSAs) as a successful and sustainable mode. Funding health through HSAs has proven to be one of the great success stories engineered by policy makers in Singapore [31]. The city state was ranked number “6” by the WHO report 2000 despite the fact that its total expenditure on health before the year 2000 did not exceed 2000 USDs per capita per year. More recent studies of Indiana state introduction of HSAs [49], showed significantly higher coverage rate as compared to Kansas, which did not expand Medicaid, during 2017. Furthermore, it showed that among low-income non-elderly adults who were familiar with HSAs, only 9% of them reported that they have been locked out of health care coverage as a result of premium non-payment. Non-pooling of HSAs and the requirements of individual consent before using the funds render HSAs resilient to financial irregularities and/or economic downturns much more than the case with pooled and/or public funds. For these reasons the degree of penetration of HSAs as a funding mechanism for health systems was selected as indicator for sustainability (indicator #8). For countries that have not adopted the HSA financing model, it is proposed to consider the household's health expenditures in relation to its annual net income. If they are less than 5%, a score of 3 will be assigned. We included policyholder's share of health insurance costs like contributions levied on the total gross income or health insurance premium (basic and supplemental) and deductibles unlike the definition of *out of pocket payments* provided by WHO [50]. We preferred to use the Delphi survey for countries for which we could not find sufficiently detailed data to estimate household health expenditures and/or the proportion of wage deducted directly for health insurance. There may be a positive correlation between health expenditures and the life expectancy of a population, but this is not always the case, which is why we have distinguished it from indicator #2. Gains in life years depend not only on the size of health expenditures, but also on the way in which they are used and distributed [51]. The United States is a good counter-example, since it spends more on health care than other countries but has a lower life expectancy than other countries that spend much less.

The rationale selection of these indicators can be subject to criticism because it can be considered as the judgement of individual researchers only. As a neutral public academic institution, no indicator has been selected to favor one country over another. If the selected indicators do not seem to have a specific value that would make them more usable than (any) other single indicator, we believe that the combination of these nine indicators will allow a rapid overall assessment of the performance of a health system in terms of sustainability, quality, access or clinical outcomes.

The objective in proposing this alternative ranking system is to design, implement, monitor and reform sustainable health systems to enable them to meet the challenges of the disease burden at national and global levels. This proposed framework aims to serve more countries, including low- and middle-income countries, although it could be criticized that these countries have not been selected on our framework. We assumed that it would be more difficult to assess them because of the limited data availability and accessibility in settings with restricted health care resources, particularly because of difficulties in defining common performance indicators or providing standardized data collection mechanisms. Access and sharing of accurate and relevant data concerning developing countries remains a major challenge for the years to come in term of financial, technical and human resources [52,53]. Our framework was developed on the principle that the evaluation of a health system's performance should be based on the same criteria of excellence, independently of its standard of living (low-middle-high income countries). We recognize, however, that the economic dimensions of health systems in low-income countries are different from those in high-income countries, which inevitably impacts on their health policy priorities. For example, high-income countries are more concerned with containing costs while maintaining high-quality services with high technology, while low-income countries are more concerned with increasing geographic coverage and the supply of basic services in a context of high needs and minimal financing [54]. We believed that despite this, low- and middle-income countries can benefit from this framework in order to implement appropriate public health intervention according to their settings [55].

Social determinants of health influence the performance of a health system (eg, availability of resources to meet daily needs, socioeconomic conditions, culture [56]. However, we have decided not to include them in our ranking. We considered them as simply beyond the control of a health system and believed that they should be considered independently of the health system being evaluated. It may indeed be difficult to disentangle them and assess the extent to which they are the responsibility of a health system. For example, if the culture has a predilection for unhealthy foods and this impacts on the occurrence of chronic disease, there may be little that health care providers can do about it. Conversely, if the culture has a pre-existing preference for healthy foods, the health care system does not deserve to be rewarded either [17]. Only indicator #2 incorporates the issue of gender equity by evaluating HAQ among female and not both genders.

It is also true that this framework integrates the evaluation of chronic disease management (eg, Hodgkin's disease; cardiovascular disease) to a greater extent than that of communicable diseases. We though that the recording of communicable diseases could be under-evaluated, especially in low-income countries due to insufficient diagnostic capacity, as it is the case with COVID-19 [57]. In the context of the emergence of chronic diseases - in low- and middle-income countries, we preferred to prioritize this measure as well.

The ranking presented here will evolve over time with the acquisition of more data that will be able to better ascertain the score for each missing indicator as we consider that that framework can be subject to re-consideration of one or more of the indicators used should scientific evidence support such an undertaking. We believe that many data should exist but are not always published and shared as an open source data. It is also sometimes difficult to find data simply because of language restrictions or difficulties in accessing them, especially on government sites.

The Delphi survey can also be objected because it has methodological limitations with a low level of evidence and high dependence of experts’ opinion. It also had very few participants. However, results of our survey don’t intend to replace any factual data sources, only augmented preliminary estimates until more accurate data will be shared. As WHO mentioned in its 2000 report, effort should be made to get and publish more and better data on all core population health indicators in an officially way. We think that such rating can help in the evaluation of Health system’s agility and response to major disasters such as COVID-19.

Other potential indicators we consider worthy of further evaluation include human capital index, compliance with International Health Regulations, future health scenarios and findings from the national commissions on high quality health systems [58,59].

We hope that these preliminary data will prompt the competent authorities to report more accurate values for these indicators but also strengthen their means to obtain them if they are not available. That’s why we welcome individual Countries who wish to be included in this and future Ranking System reports, or indeed those countries who wish to update or correct one or more score value(s) for performance index (indices) presented. The Institute of Global Health, Faculty of Medicine, University of Geneva, Switzerland will support holding a database solely for this purpose and will generate an annual International Health System Ranking report using data provided by a formal communication from individual Ministry of Health, or respective Mission to the UN in Geneva to the following email address: Antoine.Flahault@unige.ch. To guarantee data quality, please note that only data provided through official Ministry’s or Mission’s email address will be considered for this task.

## CONCLUSIONS

This ranking system is modeled to test health systems’ outcome performance and robustness and to guide “various organizations, people and actions whose primary intent is to promote, restore or maintain health” to better design, implement, monitor and reform sustainable health systems poised to meet the challenges of the burden of disease at National and Global levels.

While it is true that data are still missing, we hope that this work will encourage ministries of health to get and publish more and better data on all core population health indicators in an officially way. The emergence COVID-19 is a reminder of how a sustainable health system, together with appropriate mitigations, through early detection, isolation and adequate health services across its three levels can reduce the impact such an overwhelming Pandemic on individual countries and the World. Further research is required to test the applicability of our ranking system and compare it to the WHO’s and/or other ranking systems.
